# Novel smart window using photonic crystal for energy saving

**DOI:** 10.1038/s41598-022-14196-9

**Published:** 2022-06-16

**Authors:** Zaky A. Zaky, Arafa H. Aly

**Affiliations:** TH-PPM Group, Physics Department, Faculty of Science, Beni-Suef University, Beni-Suef, Egypt

**Keywords:** Photonic crystals, Nanophotonics and plasmonics, Sensors and biosensors

## Abstract

Smart windows are emerging as an effective way of minimizing energy consumption in buildings. They attracted the major relevance for minimizing energy consumption in buildings. More research studies are needed to design smart windows with operating wide range and don’t require additional energy to operate. We suggest a novel smart window structure using photonic crystal to regulate the solar radiation intensity by preventing it from penetrating the buildings in summer. For the first time, the suggested smart window photonic crystal at room temperature is proposed. The suggested smart window can block about 400 nm of near-infrared. This smart window model doesn’t require additional heat or electric input to operate.

## Introduction

Switchable electromagnetic wave transmittance using smart windows has recently attracted attention because they are involved in skylights, architectural or vehicle windows, and internal partition applications^1^. Because commercial and residential buildings consume around 40% of overall energy consumption, the implementation of smart windows is of major relevance for minimizing energy consumption in buildings^2^. Heating, air conditioning, and ventilation account for more than half of the total energy consumption^3^.

Smart windows control how much ultraviolet (UV), visible, and, most importantly, near-infrared (IR) radiation penetrates buildings. Inside facades, blinds can be used to regulate the solar radiation intensity by preventing it from penetrating the building in summer to reduce the cooling costs. The intercepted solar energy in this traditional way is not being used. Even though Photochromic, electrochromic, and thermochromic materials are chromogenic materials that have been widely used for smart windows to conserve energy, they require additional heat or electric input to operate^4–6^.

The dispersion of electromagnetic waves through periodic structures is greatly affected by the photonic band gaps (PBGs). Recently, photonic crystals (PCs) are used in different applications such as filters, biosensors, solar cells, etc.^7–23^. Currently, some advanced spatial smart windows are implemented using the spatial propagation features of traditional PCs^24^. Arafa et al. in 2019 presented a low-temperature smart window using superconductor PC^25^. Blocking or passing the electromagnetic waves in this structure is controlled by changing the incident angle. Also, it can’t be used for our buildings because it depends on the usage of superconductor material. It can be used only for low-temperature applications. In 2020, Arafa et al. proposed an optical filter structure to control the propagation of light through it depending on the incident angle^26^. Besides, this structure can’t record high (for winter) and low (for summer) transmitted infra-red light at the same time.

The current work is distinct in some ways. For the first time, a detailed simulation study of smart window PC at room temperature is proposed. Moreover, the proposed structure has the ability to block a wide range of IR by filling the air layers with water. It can work at a long range of incident angles. Moreover, the suggested structure doesn’t require additional heat or electric input to operate.

## Basic equations and materials

**Figure 1:**
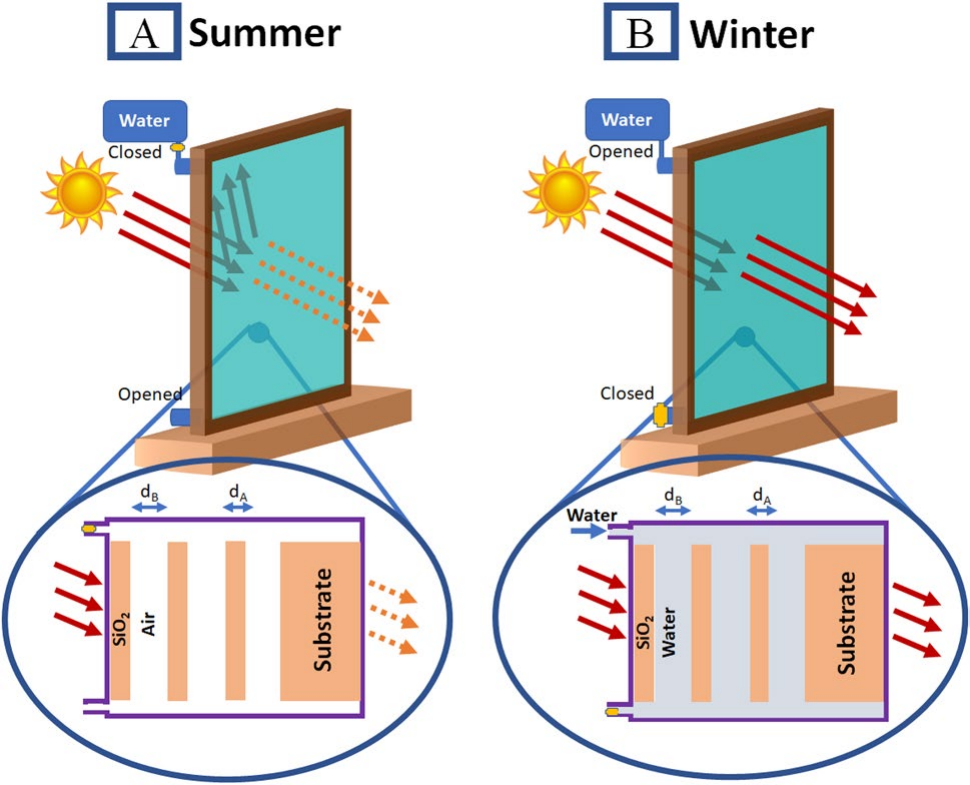
Schematic diagram of proposed smart windows (**A**) in summer, visible electromagnetic waves will be transmitted and IR will be blocked, (**B**) in winter, visible and IR electromagnetic waves will be transmitted.

Figure 1 presents the proposed smart windows. In the case of Fig. 1A, the visible electromagnetic wave will be transmitted, and IR will be blocked. It is composed of (SiO_2_/Air)^N^ photonic crystal. By filling the cavity layer with water, visible and IR electromagnetic waves will be transmitted as clear in Fig. 1B. N clarifies the number of times the photonic crystal periods will be repeated. The proposed smart windows structure will be deposited on SiO_2_ substrate.

SiO_2_ layers can be deposited using different techniques such as ion assistance electronic beam deposition^27^ and radiofrequency magnetron sputtering^28^. The refractive index of SiO_2_ can be calculated by using the following fitted equation^27^:

1 $${\text{n}}_{SiO2} \left( \uplambda \right) = 0.0137 \uplambda^{6} - 0.1286 \uplambda^{5} + 0.4855 \uplambda^{4} - 0.9457 \uplambda^{3} + 1.0055 \uplambda^{2} - 0.5600 \uplambda + 1.5974,$$

where ${\uplambda }$ is the wavelength in µm. We use SiO_2_ as a dielectric material because the imaginary part is close to 0 and can be ignored^27^. Zaky et al.^11^ cleared a method that can help to control the thickness of Air/water layers during the process of fabrication using the etching process. For certainty, we assume d_A_ = 175 nm, d_B_ = 270 nm, N = 3, n_Air_ = 1, n_water_ = 1.33^29^, and $\theta_{0} =$ 0 degrees as initial conditions.

As the incident sunlight is completely unpolarized, each polarized component (TE and TM components) has its own proper behavior inside the proposed design^30^. The transmittance of the proposed smart windows for TE and TM electromagnetic waves will be calculated by the transfer matrix method (TMM) to investigate how the proposed smart windows react to incident waves^31–34^:

2 $$T\left( \% \right) = 100*\frac{{p_{0} }}{{p_{s} }}\left| t \right|^{2} ,$$

where

3 $$t = \frac{{2p_{s} }}{{\left( {A_{11} + A_{12} p_{0} } \right)p_{s} + \left( {A_{21} + A_{22} p_{0} } \right)}} ,$$

4 $$\left| {\begin{array}{*{20}c} {A_{11} } & {A_{12} } \\ {A_{21} } & {A_{22} } \\ \end{array} } \right| = \left( {a_{SiO2} a_{Air/water} } \right)^{N} ,$$

5 $$p_{k} = n_{k} cos\left( {\theta_{k} } \right)\;{\text{for}}\;{\text{TE}}$$

6 $$p_{k} = \frac{{cos\left( {\theta_{k} } \right)}}{{n_{k} }},\;{\text{for}}\;{\text{TM}}$$

where $\theta_{k}$, $n_{k}$, $a_{k} ,$ $p_{0}$, and $p_{s}$ are the incident angle of the electromagnetic waves, the dielectric constant, the transfer matrix element, $p_{0} {\text{and }}p_{s}$ related to air and substrate.

### Ethics approval and consent to participate

This article does not contain any studies involving animals or human participants performed by any of the authors.

## Results and discussions

To demonstrate the capability of our smart window structure to control the propagation of IR range, we conducted a series of simulation studies using TMM.

**Figure 2:**
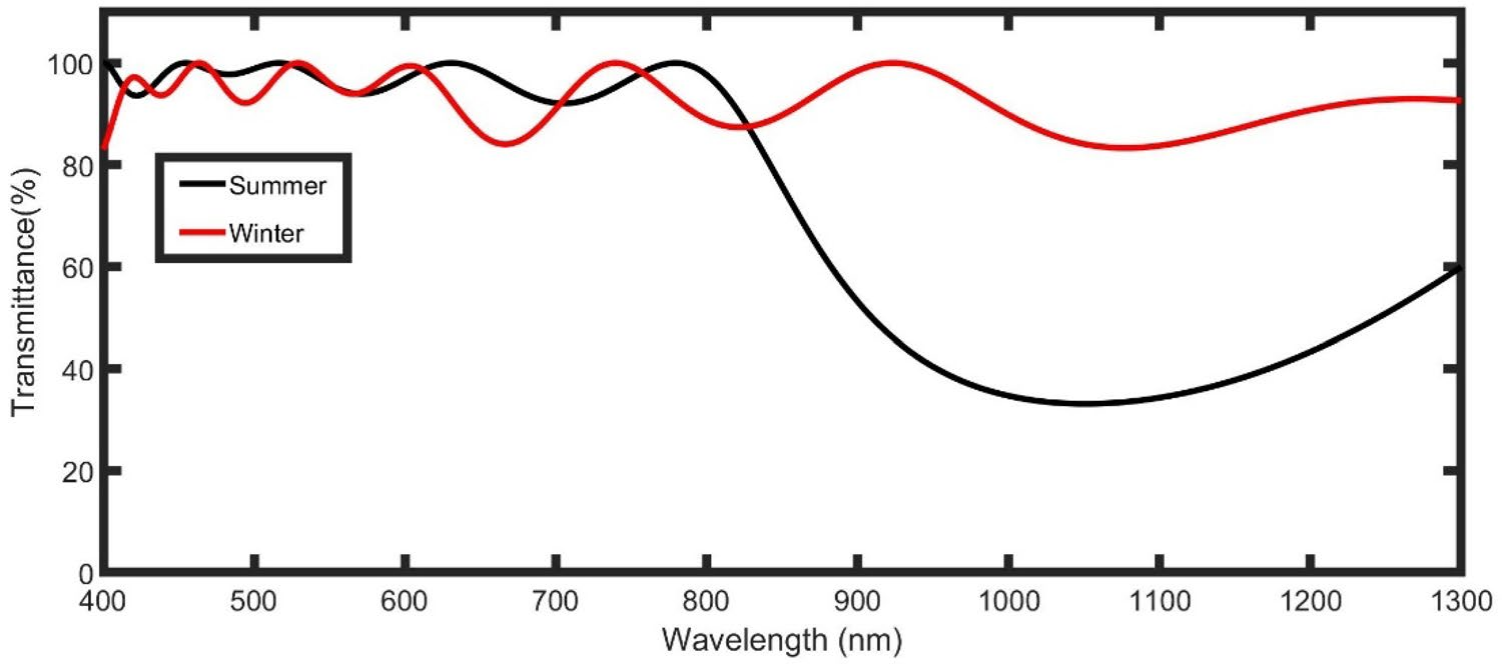
The transmittance of the proposed smart windows for TE polarized light at initial conditions.

For this study, we need a window that lets the visible and near IR range propagate through in winter to warm the buildings internally, and blocks IR in summer. A structure with two conditions will be used for these states, as clear in Fig. 1. In the event that the structure layers are (SiO_2_/Air)^N^, there is a high refractive index contrast between n_SiO2_ and n_Air_. As a result, a PBG appeared as clear in Fig. 2 (black line). Through the PBG, the intensity of the transmitted IR decreases and reaches a minimum value of 33% at the wavelength of 1053 nm.

By filling the air layers with water as clear in Fig. 1B, the refractive index contrast between the two used layers (n_SiO2_ and n_water_) strongly decreases. So, the PBG disappeared at the range of wavelength of concern, as clear in Fig. 2 (red line). Then, the structure allows both visible and IR to propagate through it with high transmittance ranging from 90 to 100% and warm the buildings.

**Figure 3:**
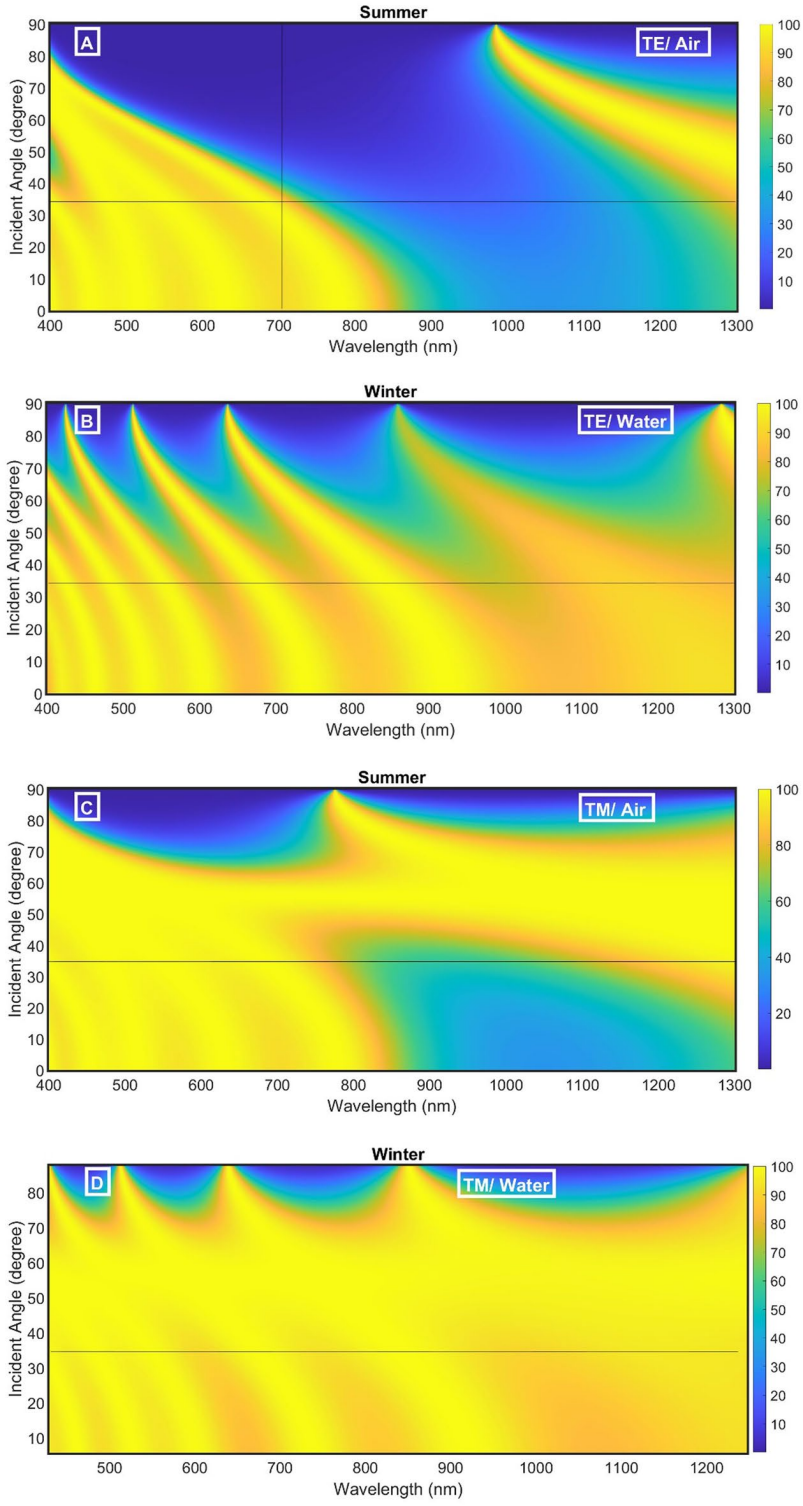
The transmittance of the proposed smart windows at initial conditions as a function of the incident angle (**A**) TE/Air, (**B**) TE/water, (**C**) TM/Air, and (**D**) TM/water.

In the following studies, we will try to reduce the intensity of IR to achieve a complete blocking as possible as we can. The effect of the incident angle of TE and TM polarized light on the PBG is studied in Fig. 3 for both two conditions (n_SiO2_/n_Air_ and n_SiO2_/n_water_). In order to assess the impact of the incident angle on PBG, the transmittance of the structure versus the wavelength at different incident angles was studied for both TE and TM polarized light.

As clear in Fig. 3A, for the summer structure, the PBG is shifted to lower wavelengths with increasing the incident angle of TE polarized light according to the following relation^35,36^:

7 $$P = m\lambda = n_{eff} G,$$

where P is the optical path difference, m is an integer, $n_{eff}$ is the periodic structure's effective dielectric constant, w is the incoming light wavelength, and G is the geometrical path difference. Since the PBG at the angle of 35 degrees extended from 700 to 1300 nm with a width of 600 nm, this angle is very suitable for the proposed application. Besides, at this angle, a moderated PBG extended from 800 to 1150 nm with a width of 350 nm for TM polarized light, as clear in Fig. 3C.

For winter structure, the angle of 35 degrees also records a suitable visible and IR transmittance for TE polarized light, and high transmittance in the case of TM polarized light, as clear in Fig. 3B,C. So, we recommend this angle in the following studies.

**Figure 4:**
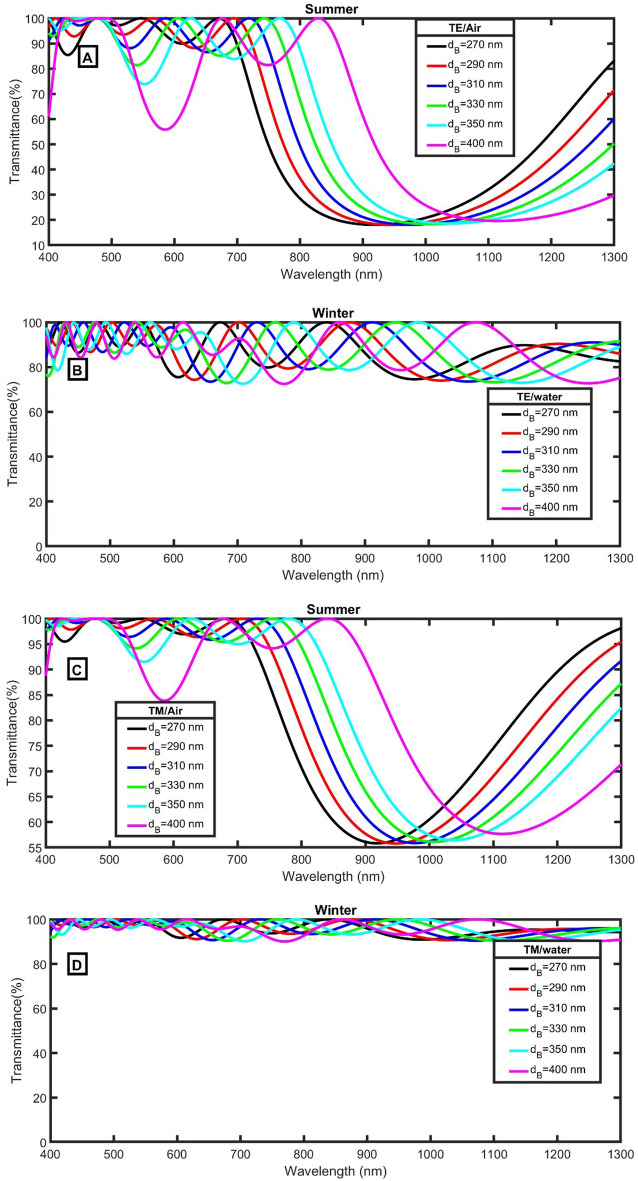
The transmittance of the proposed smart windows at 35 degrees and different values of d_B_ (**A**) TE/Air, (**B**) TE/water, (**C**) TM/Air, and (**D**) TM/water.

Because the PBG is proportional to the thickness of the used materials, the PBG shifts to higher wavelengths for higher thicknesses of the air/water layer according to Eq. (7). As clear in Fig. 4A,C, with the increase of thickness, the amount of IR with high energies (low wavelengths) can be transmitted for both TE and TM polarized light. So, the thickness of d_B_ = 270 nm will be recommended for the following study.

**Figure 5:**
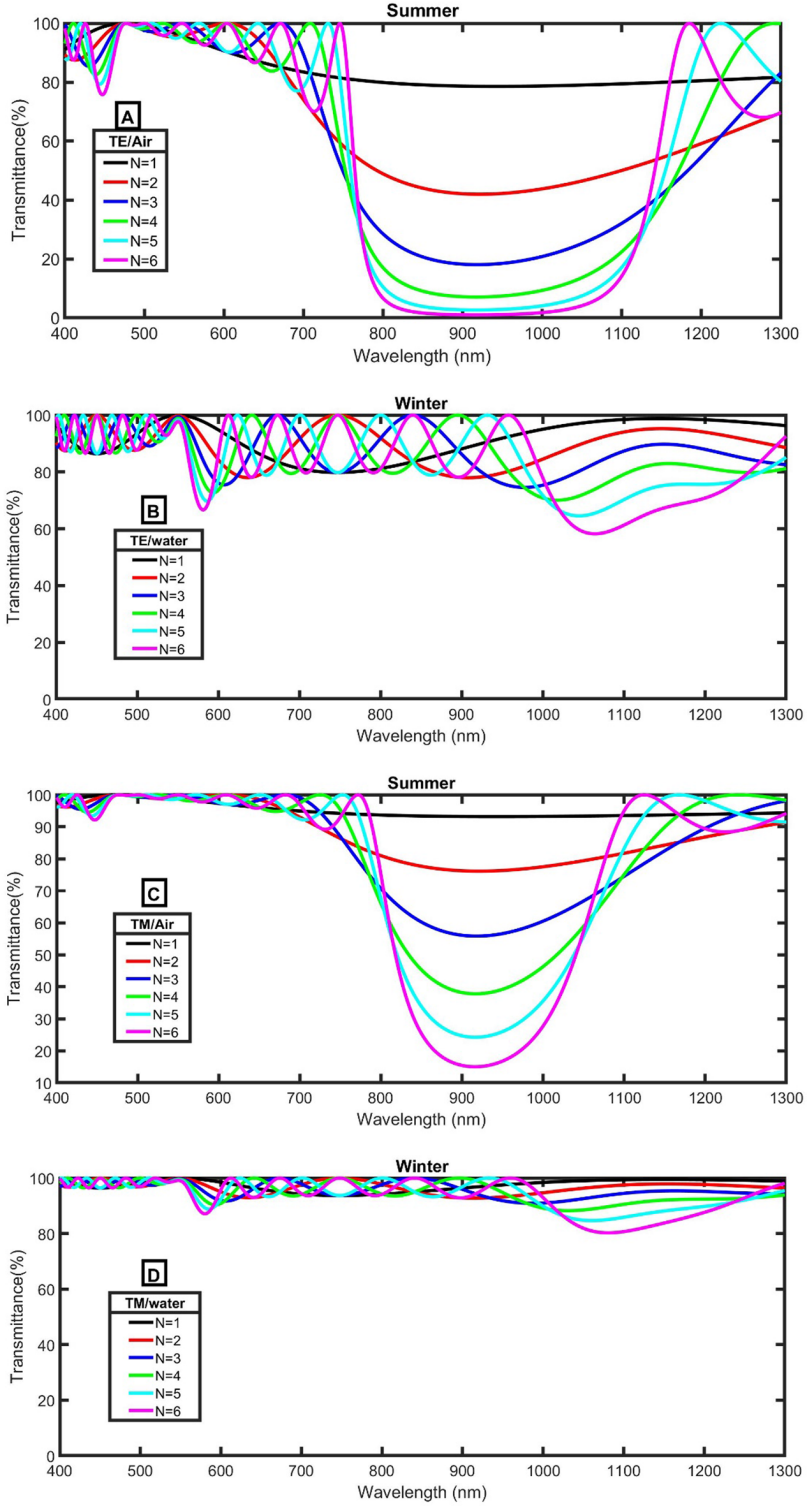
The transmittance of the proposed smart windows at 35 degrees, d_B_ = 270 nm and different values of N (**A**) TE/Air, (**B**) TE/water, (**C**) TM/Air, and (**D**) TM/water.

In Fig. 5, the number of periods will be changed but all other conditions are constants. For the summer structure, the center of PBG seems to be at the same position but the width of the PBG increases and the transmittance decreases. Besides, the edges of the PBG become more vertical and are slightly shifted to higher wavelengths. On the other hand, for winter structure, the transmittance of both TE and TM polarized light slightly decreases.

Where N = 6 has the ability to block much amount of IR in summer, it will be recommended after increasing the incident angle to 40 degrees to adjust the left edge of the PBG at the end of the visible light range and the beginning of IR range. Besides, the transmittance of unpolarized sunlight (blue line) is calculated according to the following equation^30^:

8 $$T_{unpolarized\;sunlight} = \frac{TE + TM}{2},$$

**Figure 6:**
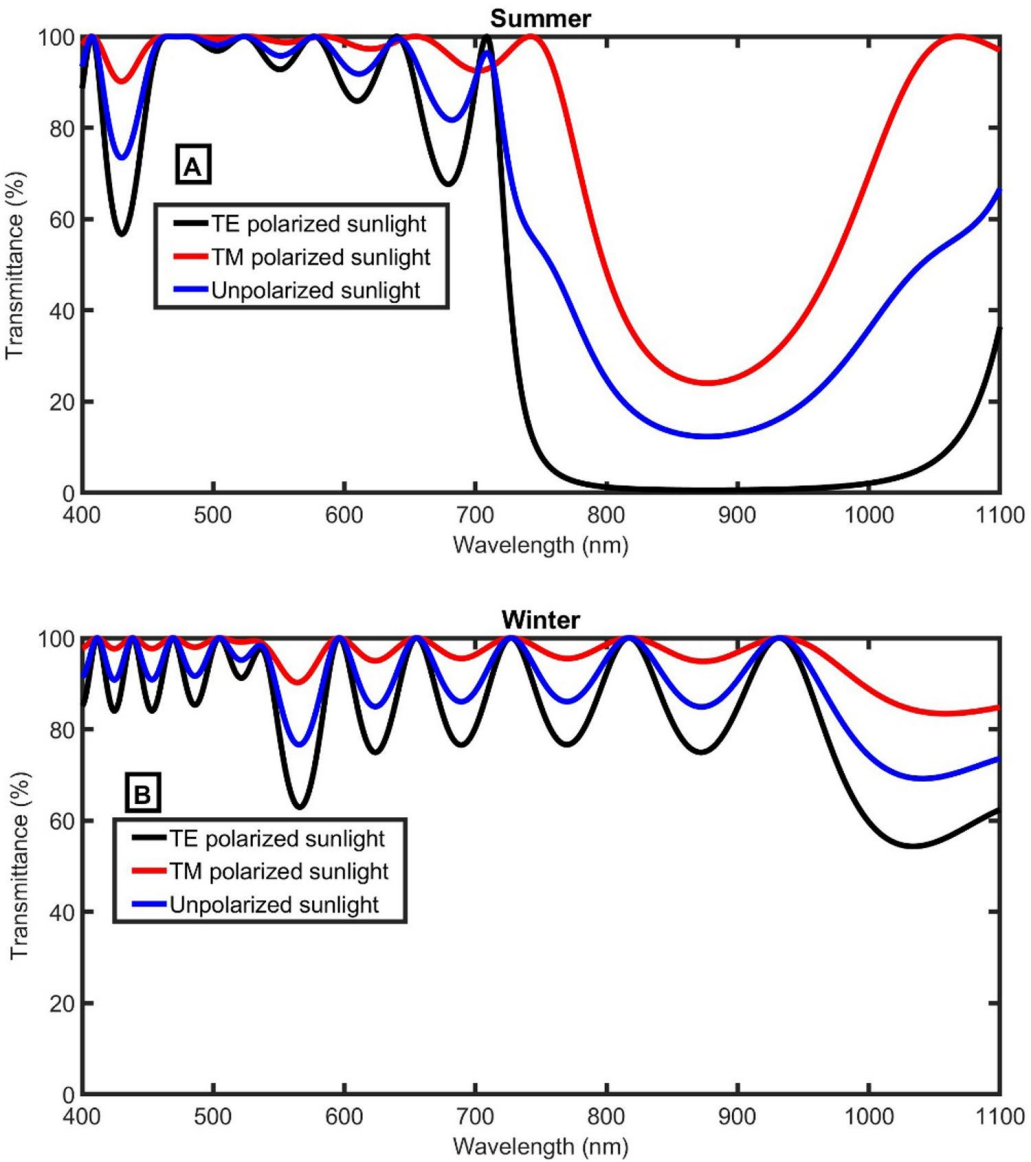
The transmittance of the proposed smart windows at 40 degrees, dB = 270 nm, and N = 6 (**A**) in Summer, and (**B**) in Winter.

It is worth noting that the efficiency of the proposed smart window for blocking TE polarized light is higher than TM as clear in Fig. 6A,B.

## Conclusion

We have utilized TMM simulation studies on 1D-PC as a novel smart window at room temperature. This structure recorded high efficiency for blocking a wide range of IR. The results cleared the performance of the proposed smart window and its potential for energy consumption. However, as much or more work remains to be done to improve the efficiency of smart windows to be able to block all ranges of IR and UV for indoor comfort and energy saving.

## Data Availability

The datasets used and/or analyzed during the current study are available from the corresponding author on reasonable request.
